# Automated Video-Based Capture of Crustacean Fisheries Data Using Low-Power Hardware

**DOI:** 10.3390/s23187897

**Published:** 2023-09-15

**Authors:** Sebastian Gregory Dal Toé, Marie Neal, Natalie Hold, Charlotte Heney, Rebecca Turner, Emer McCoy, Muhammad Iftikhar, Bernard Tiddeman

**Affiliations:** 1 Department of Computer Science, Aberystwyth University, Aberystwyth SY23 3DB, Ceredigion, UK; seg22@aber.ac.uk (S.G.D.T.); ifi@aber.ac.uk (M.I.); 2 Ystumtec Ltd., Pant-Y-Chwarel, Ystumtuen, Aberystwyth SY23 3AF, Ceredigion, UK; marie@ystumtec.co.uk; 3 School of Ocean Sciences, Bangor University, Bangor LL57 2DG, Gwynedd, UK; n.hold@bangor.ac.uk (N.H.); c.heney@bangor.ac.uk (C.H.); b.turner@bangor.ac.uk (R.T.); mrm21fgz@bangor.ac.uk (E.M.)

**Keywords:** computer vision, frame selection, keypoint detection

## Abstract

This work investigates the application of Computer Vision to the problem of the automated counting and measuring of crabs and lobsters onboard fishing boats. The aim is to provide catch count and measurement data for these key commercial crustacean species. This can provide vital input data for stock assessment models, to enable the sustainable management of these species. The hardware system is required to be low-cost, have low-power usage, be waterproof, available (given current chip shortages), and able to avoid over-heating. The selected hardware is based on a Raspberry Pi 3A+ contained in a custom waterproof housing. This hardware places challenging limitations on the options for processing the incoming video, with many popular deep learning frameworks (even light-weight versions) unable to load or run given the limited computational resources. The problem can be broken into several steps: (1) Identifying the portions of the video that contain each individual animal; (2) Selecting a set of representative frames for each animal, e.g, lobsters must be viewed from the top and underside; (3) Detecting the animal within the frame so that the image can be cropped to the region of interest; (4) Detecting keypoints on each animal; and (5) Inferring measurements from the keypoint data. In this work, we develop a pipeline that addresses these steps, including a key novel solution to frame selection in video streams that uses classification, temporal segmentation, smoothing techniques and frame quality estimation. The developed pipeline is able to operate on the target low-power hardware and the experiments show that, given sufficient training data, reasonable performance is achieved.

## 1. Introduction

### 1.1. Background and Motivation

There is a moral and legal obligation for countries to manage their fish stocks sustainably. For example, the European Union’s Common Fisheries Policy legislates for all fish stocks to be managed at an exploitation level consistent with the Maximum Sustainable Yield (MSY) [1]. The UK Fisheries Act 2020 requires stocks to be harvested to a level where biomass is above those capable of producing the MSY. In the USA, the Magnuson-Stevens Fishery Conservation and Management Reauthorization Act of 2006 requires annual catch limits and measures to end overfishing. The UN Sustainability Goals constitute 14 aims to conserve and sustainably use the oceans, sea and marine resources for sustainable development.

There has been progress in the sustainable management of fisheries, but this is mostly confined to the places where there have been the resources to undertake quantitative stock assessments and management programs, with many of these data-rich stocks fished sustainably [2,3]. However, in fish stocks that are data poor or not intensively managed, many are still considered unsustainable [4,5] and it is estimated that over 80% of global stocks are not formally assessed [4]. This, therefore, drives the desire to maximise data collection to support quantitative stock assessment that can support management. There are a wide range of fisheries stock assessment methods, each of which can have varying data input needs.

Crustacean fisheries are important globally, accounting for over 5.5 million tonnes of capture fisheries annually [4] and landings of crustaceans have been increasing globally at a greater rate than any other capture fisheries group [6]. Whilst this is still modest in terms of overall global landings, ∼7.1%, ref. [4] crustaceans are often valuable and represent a disproportionate proportion of the value of global capture fisheries (21%) [6]. Within the UK, crab and lobster fisheries are some of the most fished and valuable species. In 2021, across the UK as a whole, crabs were the third most valuable fishery and lobsters the fifth most valuable, even though by tonnage they were less important. At a regional level they can be of even greater importance. For example, in England, crabs are the third largest fishery in terms of tonnage and the second most valuable, and lobster, whilst small in terms of tonnage was the third most valuable. In Wales, there is a similar situation, with lobster being the second most valuable and crab the fourth most valuable fishery [7]. The importance of these fisheries has been highlighted with their inclusion as “front runner” species for the development of Fisheries Management Plans (FMPs) following the new Fisheries Act 2020 and the development of the Joint Fisheries Statement.

Currently, crab and lobster fisheries in the UK are considered data poor and have limited management, mainly consisting of a minimum legal landing size. Improving the data collection for these species will be a key step in improving their management. Due to the difficulty in ageing crustaceans, many assessments focus on length-based approaches [8,9], therefore the collection of length data is often of high priority for crustacean fisheries management. For this to be representative of the whole stock, good spatial coverage is necessary and, due to changes in catchability due to biological processes such as molting, time series data across the year is also important [10,11]. However, the collection of these data often require scientific observers to be onboard fishing vessels, which is resource and time expensive, as well as having poor coverage both spatially and temporally. An alternative is the regular sampling of the landed catch at port; again, this is resource intensive, with poor spatial and temporal coverage. In addition, it only gathers information on the landings not the whole catch. Electronic monitoring has been gaining traction over the last two decades with the potential to provide cost-efficient data to supplement existing, traditional data collection programs for effort monitoring, catch composition, bycatch and gear modification [12]. The benefits of these programs are the high level of spatial and temporal coverage that can be achieved, cost efficiency and the high level of representation across the fleet [12,13]. This can be particularly useful in data-poor fisheries where there are limited resources for widespread observer programs or fishery-independent surveys.

The potential for a sentinel fleet to gather this data through the use of video data methods has previously been investigated [13], but for the cost efficiency to be maximized, the automation of the extraction of the catch composition data is required.

The system we have developed provides a reliable, low-cost solution that is able to operate in challenging conditions. It provides a template that could be adapted for similar problems in fields such as biology, ecology and marine research. A key novel contribution of the developed pipeline is a method to extract a single frame, or a small set of frames offering different key views, of each animal. Without this component, the system would “over count” each animal, providing multiple measurements of the same animal. Even in high-powered systems using state-of-the-art (SOTA) object detectors, object detections (locating an object within a scene) over time are noisy, and distinguishing genuine breaks between different instances of the same object vs temporary detection loss is challenging. The problem is even more challenging in a setting with limited computational resources. Following frame selection, greater computational resources can be expended on the small number of frames selected for the proceeding steps, including object localisation and point detection, needed to provide information on the size of the animals. The remainder of this paper first discusses the relevant prior research and preliminary work that informed the design of the system, then we present the system description (including the hardware and each step of the pipeline), followed by the results and discussion.

### 1.2. Related Work

#### 1.2.1. Computer Vision for Crabs and Lobsters

This work automates the video-based approach described in reference [13]. There has been considerable prior work on the detection and analysis of marine animals, particularly for fish detection, and some work investigating the detection of crabs and lobsters. Most recent systems use standard object detectors as a backbone, with some bespoke adaptations and fine tuning. For example, Cao et al. [14] use MobileNetV2 [15] as a backbone for an optimized Single Shot Detector (SSD) for underwater crab detection. Their system focused on detection (in every frame) and did not extend to temporal segmentation (for counting and avoiding repeated measurements of the same animal) or measurement. Similarly, Ji et al. [16] developed a system for detecting underwater river crabs using the MobileCenterNet model, again based on MobileNetV2, they also focused on detecting within static images, and did not address temporal segmentation issues. Chen et al. [17] developed a detector and gender classifier for Chinese Mitten Crabs based on an optimized Yolo-v4 architecture, but also did not address temporal segmentation issues. Tang et al. [18] and Hu et al. [19] have developed Yolo (v3 and v4, respectively)-based systems for detecting molting events in commercial crab breeding. Again, there was no requirement for temporal segmentation to detect individual animals. Wu et al. [20] developed a part-based model for identifying individual crabs but, in our work, there is no requirement to identify specific individual animals. Recognising individual animals is a potential way to try to avoid repeated counting/measuring of the same animal, but is an expensive approach, requiring checking of the identity in each frame, and not well suited to our low computational power scenario.

Mahmood et al. [21] used synthetic data to train a Yolo-v3 detector for the underwater detection of rock lobsters. As we have the capability to capture a sufficient volume of data, we have not found it necessary to use synthetic training data in this work. Chelouati et al. have used Yolo-v4 [22], and more recently Yolo-v7 [23], to successfully detect lobsters in a robotic application scenario. Again, this scenario has no requirement for the temporal segmentation for animal counting/measuring addressed here.

For the sub-problem of measurement, Wang et al. have developed deep learning methods for segmenting crabs [24] and detecting specific features (knuckles) [25]. The segmentation algorithm uses a shallow U-Net style architecture. The knuckle detection used a combination of traditional approaches (e.g., background subtraction and watershed algorithm) and a deep learning point detection algorithm based on the VGG architecture. As their system is intended for processing-plant-based application, it is not subject to the same low computational processing power constraints as the work described here, but does provide a useful starting point for our work.

#### 1.2.2. Lightweight Computer Vision

Whilst many lightweight models are proposed in the literature for low-power computer vision applications and edge computing, it was found that many of these still benefited from relatively powerful hardware. Most commonly, an Nvidia Jetson Nano 4GB Kit was chosen for real-time detection applications [26,27,28]. In the few studies in which a Raspberry Pi was used, it would typically be a Raspberry Pi 4 4GB model [28,29]. The effects of the semiconductor shortage at the time of writing have contributed to the increased cost of these devices, as well as the lack of availability for the more powerful and, therefore, more popular Single Board Computers (SBCs). Given that real-time processing is not essential for this work and this project has a constraint on power-usage, the less powerful Raspberry Pi 3A+ was chosen for its lower cost and better availability. One other consideration was that the device must be contained within a waterproof enclosure where active cooling using fans would not be effective. Choosing more powerful hardware such as the Nvidia Jetson devices may have led to overheating issues. Whilst processing power and, thus, inference time, would be an obvious issue for the Raspberry Pi, the main obstacle was, in fact, that many supposedly lightweight computer vision models would not fit into the 512MB memory of the Raspberry Pi 3A+ (alongside the OS, input data and pipeline-specific structures) and failed to execute at all.

Several older, heuristic-based methods were included in the preliminary investigation [30,31,32] for the possibility of cheaper solutions given the limited computational resources. Wolf [30] describes a method of frame selection that analyses sequences of frames for relative motion. The heuristic is that stillness within the video implies the contents or subject are to be focused on. Given the use case of this system and how animals will be presented to the camera, this appears to be a perfect fit. However, the environment in which this system will be used is highly dynamic. Events such as water splashing, shadows moving, lighting conditions changing as clouds move, the boat rocking, ropes and pots being moved within the scene, etc., caused frequent false negatives for detecting stillness in our preliminary investigations. Elgammal et al. [31] propose a similar solution for background subtraction. Each frame is compared with a model of the scene background that is updated frequently and regions of the frame that do not match this model can be classed as foreground. Their solution is somewhat robust to acutely dynamic environments (tree branches swaying), by actively suppressing false detections, but relies on a static camera position. Background subtraction (and contour extraction) have been used for automated counting [33,34,35] and are shown to work reliably in highly controlled environments with specific conditions. Preliminary investigations indicated that our intended environment would exceed the ability of this suppression technique as it is highly dynamic and a sufficiently static camera cannot be guaranteed due to high-winds, the frame containing the sea over the edge of the boat and the parallax effect of the boat rocking. Finally, the well-known Viola–Jones method [32] was initially investigated as a cheap and efficient object detector. However, it is known that this method is rotationally variant (fails to detect objects when rotated) and must be ruled out because no guarantee can be made for the orientation of the presented subjects.

As none of these classical image processing methods were found to be suitable, investigation was aimed again towards learned methods that could run at a reasonable frame rate on the target hardware. Many of the recent computer vision architectures intended for edge devices leverage Depthwise Separable Convolutions [36,37] as a computationally cheaper alternative to traditional convolutional layers. Simply put, the input data are analysed ‘depthwise’ for features using 2D convolutions (width and height), one colour channel at a time. This stage is followed by ‘pointwise’ convolutions (1 × 1 pixels) that combine the feature information from each channel in the previous step. Whilst, theoretically, the number of operations overall is similar to that of traditional convolutions, breaking the problem into a greater number of smaller computations is advantageous when using edge devices. In MobileNetV2 [15], this technique is also used as a part of a ‘Linear Bottleneck’ where 1 × 1 and 3 × 3 convolutions are used to reduce the number of channels until an intermediate point, where it is then expanded, creating a bottleneck. This bottleneck is known to improve information flow through the network which aids the training process, as well as improving computational efficiency. The term ‘linear’ refers to the gradual change in dimensionality between each layer rather than an extreme difference. Another contribution presented in MobileNetV2 [15] is the ‘Inverted Residual’ block, which uses a large feature space and compresses it, contrary to traditional residual blocks found in ResNets [38]. By design, this reduction in dimensionality preserves the features of importance whilst discarding others. Each of these innovations improves efficiency for edge devices by simplifying the computation performed at each step. Another important alteration presented in MobileNetV3 [39] is the use of ‘Neural Architecture Search’ (NAS) to discover optimal hyperparameters and configurations of the previously mentioned blocks (depthwise separable convolution, linear bottleneck, inverted residual).

Additionally, an implementation of YoloV5 was found that could run on the device (when converted to tflite) for object detection but was exceedingly slow. To compromise between performance and speed, it was decided that a cheap binary classifier would be used against every input frame (taking advantage of the speed), whilst YoloV5 would only be invoked on relevant, high-quality frames that are predicted to contain an animal (taking advantage of the precision). This setup is described in further detail in Section 2 and Section 2.2.

## 2. Materials and Methods

### 2.1. Hardware Setup

The solution is aimed to be as non-intrusive as possible so that it does not impede the fishers during their work. For this reason, a camera system was chosen because this does not require any interfacing with the user and only needs them to present the animal to the camera for a brief period of time. Given that the camera will capture high-resolution video, it was deemed unfeasible to transmit these data over a cellular connection (Global System for Mobile communication—GSM) for processing on the mainland. Instead, the camera system is powered by a Single Board Computer (SBC) that processes the video on the fishing boat. After the Computer Vision system has finished processing, the resultant data values can then be transmitted to a central system more efficiently. The SBC component is based around a Raspberry Pi 3A+ which offers a low-cost and rapid route to producing a flexible prototype. Given the limited computational resources of the device, it was decided that the Computer Vision system would not run in real time. Instead, a scheduling system has been implemented that detects how fast the boat is moving from GPS data. If the boat is moving <4 knots, it is considered to be in the ‘fishing’ state and the device simply records and stores the video data. If the boat is moving ≥4 knots *or* the GPS coordinates are within a given radius of the port, then the boat is considered to be travelling or docked. In this case, the device can switch to processing the stored video data.

**Figure 1:**
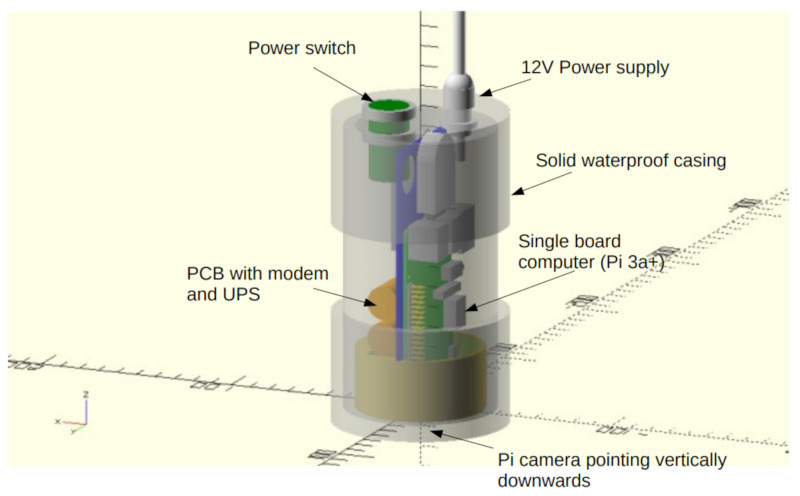
A 3D model of the device with semi-transparent housing to show internal components.

**Figure 2:**
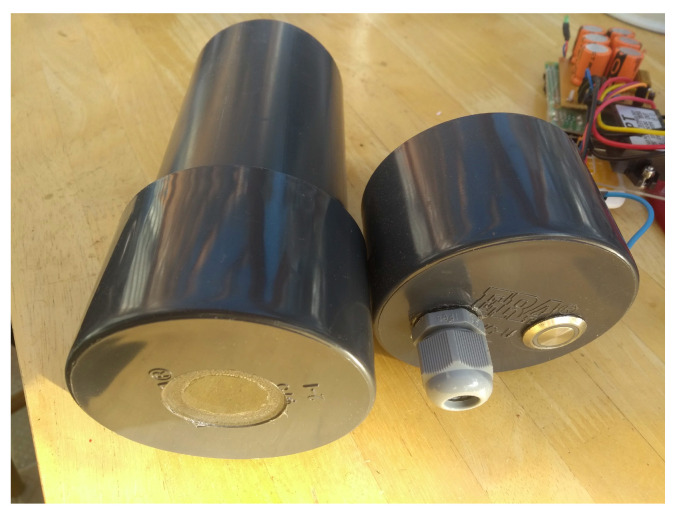
A photograph of the capture device with top part removed showing the camera window and power supply and switch.

The Raspberry Pi 3A+ includes hardware for the provision of the WiFi access point which will be used for configuration and management. In addition to the Raspberry Pi 3A+, we have designed a Pi ‘hat’ printed circuit board (PCB) which plugs directly into the main header and includes the other components of the system (GSM modem and Global Navigation Satellite System (GNSS) receiver) as well as a small uninterruptible power supply (UPS), fuse and power conditioning systems. The PCB uses a SIMCOM 7600E modem that provides the GSM and GNSS requirements via appropriate level-shifting and power provision. An integral UPS uses a bank of ‘super capacitors’ to provide approximately 60 s of runtime after power disconnection to allow a clean shutdown of the system when it is unplugged. This assists with long term reliability as it reduces the likelihood of filesystem corruption and other operating-system-related problems. The UPS has a significant in-rush current requirement (5 A @ 12 V for 500 ms) upon start-up due to the need to rapidly charge the super capacitors; this current is limited using a series resistor and rapidly reduces after the first second of connection. The continuous current requirement after initial power-up will reduce to less than 1 A after 1 min. The SBC/PCB assembly is mounted in a 3D-printed retainer to prevent movement inside the main housing tube and the antennae for the GSM and WiFi are housed internally in the main housing to reduce the risk of damage. The camera is mounted in the bottom of the case behind an acrylic window. The housing measures approximately 170–200 mm high and 90 mm in diameter. A system diagram is given in Figure 1 and a photo is provided in Figure 2.

### 2.2. Overview of Proposed Computer Vision Pipeline

**Figure 3:**
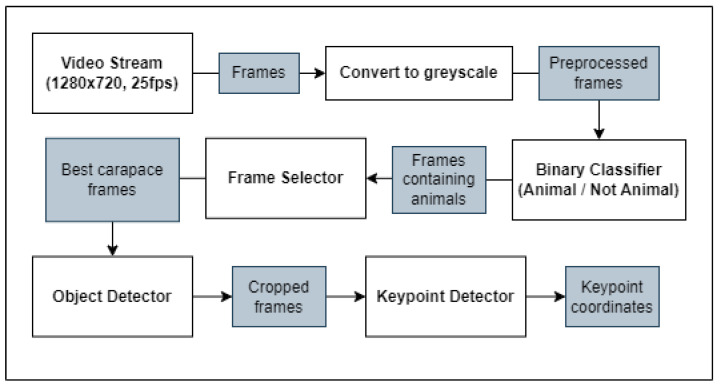
The proposed computer vision pipeline that converts a series of frames into numerical data that describes the dimensions and sex of each detected animal.

As described in Section 2.1, when a fishing boat is moving or is at the port, the onboard SBC (Raspberry Pi 3A+) is set to processing mode. Below, Figure 3 presents the pipeline through which the stored video data will be sent during this phase. The first stage is temporal segmentation, where each segment of video containing a single animal is identified—we call these contiguous segments ‘contigs’. To achieve this, a lightweight binary classifier, trained to predict whether a frame contains an animal or not, is used to filter out all of the empty frames. Each frame now has an associated prediction value, producing a ‘signal’ that spans across time. This signal is analysed to reveal where a single animal enters and leaves the scene (a contig), ensuring that the animal is counted only once. Next, the highest quality frame is selected from each contig to be used for further processing. An Object Detector is applied to each of the selected frames to find a bounding box around the animal and identify the species (crab or lobster in this case). The bounding box is used to crop the image to the region of interest (ROI) and, finally, a Keypoint Detector is used to predict the x,y position of learned features on the animal (e.g., left_eye, right_eye, tail_end, etc.). In future work, these keypoints will be used to infer the dimensions of each animal using common photogrammetry techniques, though that will not be covered in this paper.

As the keypoints will be used to derive measurements, it is important that the resolution of the input images is high to retain precision. It is also crucial that none of the preprocessing techniques distort the shape of the images used as input to the Keypoint Detector because any measurements derived from them will be equally distorted. Above all, this means that the input images cannot be resized as this would alter the object proportions. However, this level of detail is not required for the other processing stages, such as temporal segmentation and frame selection. To reduce computational cost, the high-resolution frames are resized appropriately for these specific models. Similarly, all frames are converted to greyscale at the beginning of the pipeline as this reduces the input size by a factor of 3.

The camera system records at 25 fps, meaning a huge volume of the input data consist of frames where no animal is present. This is the motivation for employing a lightweight and computationally inexpensive binary classification model, rather than processing all of these frames using the Frame Selector or Object Detector (comparably expensive models). Due to this, the Frame Selector can instead be focused on learning the concept of representativeness (frame quality, defined in Section 2.5), which benefits from a more sensitive architecture, as it does not need to learn detection.

### 2.3. Data Preprocessing and Augmentation

**Camera Data:** As described in Section 2.2, the camera system records relatively high-resolution images, with more pixels than are required for many of these computer vision tasks. The preliminary experiments of this work showed that a 1280 × 720 image could be reduced to 320 × 180 before the performance of the animal-detecting Binary Classifier (MobileNetV3-small) was affected. Of course, this aspect is determined by the camera setup and how large the subject is relative to the frame, as down-scaling will eventually render the subject unrecognisable.

Furthermore, converting the images to greyscale was found to improve inference time (fewer data to process) whilst having no effect on performance.

**Training Data and Augmentation:** In addition to the down-scaling and grey-scaling described previously, each image in the training data was augmented in the following way, based on reference [40]:

1. Randomly flip the image horizontally with probability 0.5;
2. Randomly flip the image vertically with probability 0.5;
3. Randomly blur the image with probability 0.3;
4. Randomly shift all pixel intensity values +/−20% with probability 0.3
  (simulates varied lighting conditions).

Each augmentation is applied sequentially with the given probabilities (uniform distribution), so it is possible for an image to have all augmentations applied to it (2.25% chance). Note that no augmentation techniques were applied to the test data.

### 2.4. Animal Segment Detection

In this processing stage, temporal segments of the incoming video containing a single animal are identified. Each animal should only be counted and measured once, but is present in multiple neighbouring frames. The fishers are asked to present each animal to the camera as they are removed from the pots or lines. We assume that each animal comes into the frame and leaves again, just once. The computational cost of individual animal re-identification would be too high for the intended application, and some small errors in the counting and measurement are acceptable, provided the overall statistics are reliable. Instead, a combination of binary classification and temporal filtering is used.

Of the binary classification models trialled (see Table 1), MobileNetV3-small [39] was found to have the best inference time by far. MobileNet-V1 [36] simply used too much memory for the target device and crashed on initialisation. TripleNet-S [29] was able to load without issue and could perform a small number of inferences (6 samples on average) before crashing due to lack of memory. EfficientNetB0 [41] was able to run without issue and had the best memory footprint of any model, though was not fast enough to be considered for this application. Note that EfficientNetB0 makes use of ‘inverted bottleneck’ blocks, a variation of the inverted residual layers and linear bottlenecks shown in MobileNetV3. Both models also make use of ‘squeeze-and-excitation’ [42], a method of enhancing accuracy in CNNs with little computational cost. One possible reason that MobileNetV3-Small is performing significantly faster than EfficientNetB0 is the replacement of swish activation with h-swish, a cheaper approximation of swish (sigmoid) that uses only simple operations such as multiplication and addition. Given that the TripleNet variants all use standard ReLU activations, the model is even more expensive to run. In a scenario where sufficient computational resources were available, TripleNet-S was found to run faster than MobileNetV3-Small, however, in such a constrained setting (Raspberry Pi 3A+), the model is rendered unusable.

**Figure 4:**
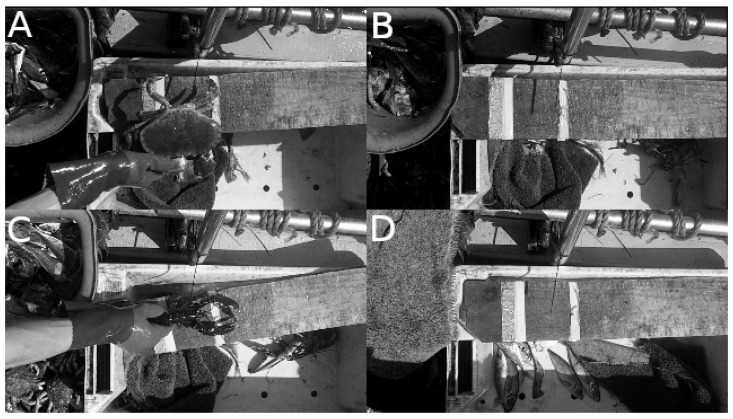
Examples of positive frames (**A**,**C**) and negative frames (**B**,**D**) used to train the classifier. The images are taken from above the processing area from a top down view. The appearance of the processing area will be different on each boat. In these examples, the animals are moved from the bucket on the left onto the horizontal plank for imaging. The boat’s railings and some tackle can be seen across the top of the image. Animals in the bucket and in the tray below the processing area cause additional difficulties for the computer vision system.

Given these results, an implementation of MobileNetV3-small [39] was chosen for the lightweight Binary Classifier seen at the start of the pipeline in Figure 3. The model was trained on a dataset of 7k images for 200 epochs with a learning rate of 0.001. The frames were taken from sample videos recorded by the camera system aboard a variety of fishing boats and augmented according to Section 2.3. The camera system was set up to simulate the real use-case as closely as possible. In the training set, half of the images are positive samples (animal present) and half are negative (no animal present). Examples of positive and negative frames are shown in Figure 4. The testing data also comprise 7k images, however, the true class proportions are preserved (6.5% positive samples). Note that the input images are rescaled to 320 × 180 before passing into the classifier and, in order for the model to run effectively on the Raspberry Pi 3A+, it must be converted to TensorFlow Lite format for inference.

**Figure 5:**
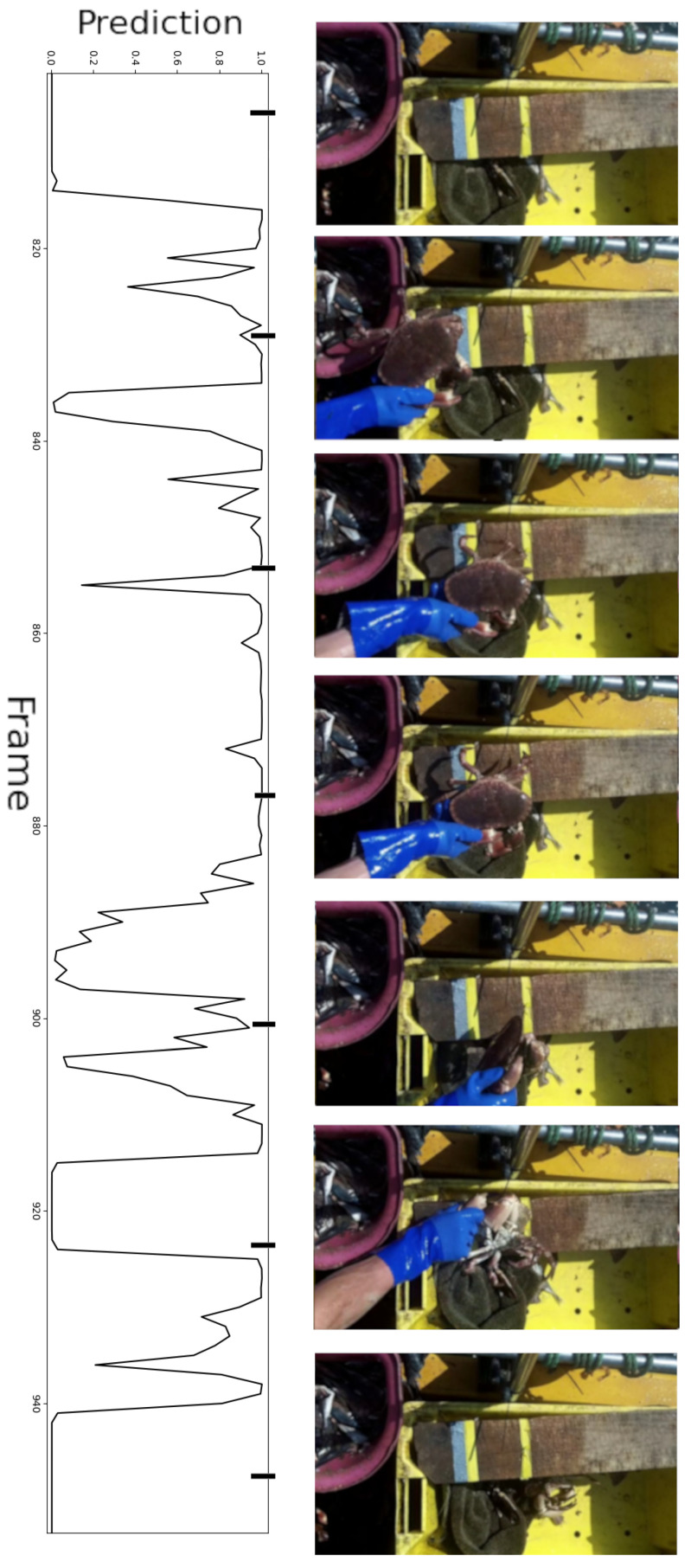
Key frames from an example video alongside the classifier response.

The drawback of using a lightweight model is, of course, the trade-off of accuracy. For single images, the classifier was found to perform well, although for continuous videos the model produced a very volatile stream of outputs, as shown in Figure 5. The animal was in the frame continuously for the majority of the video, yet the graph shows many peaks and dips. Since the limited memory of the Raspberry Pi 3A+ makes using a larger model infeasible, methods of stabilising the output were investigated.

The architecture is identical to that described for ‘MobileNetV3-Small’ in reference [39], except for the output layer. Initially, the model had one output neuron for each class (positive and negative) from which the argmax was taken, as is typical for Binary Classification. As this produced a noisy and volatile output, it was decided that a single output neuron would be used instead, leaving the value as a float between 0 and 1. This way, the stream of outputs retain the ‘confidence’ information that would otherwise be lost. Simply rounding this value would only accentuate the intermittence of the signal within a contiguous sequences of frames belonging to a single animal (‘contig’). Instead, it was found that applying a smoothing function to the entire sequence of outputs followed by the use of a step function would accurately reveal the contigs.

**Figure 6:**
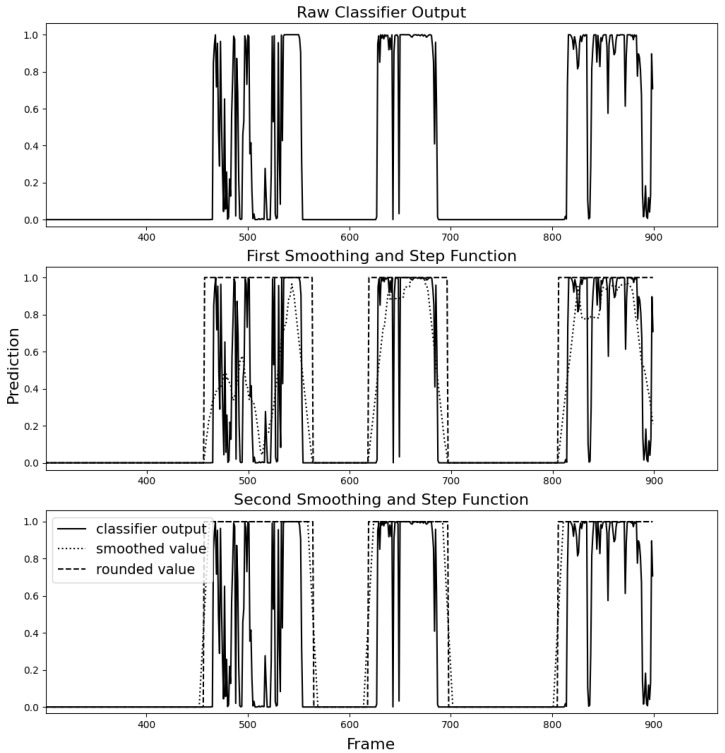
The noisy output of the classifier subject to two passes through a rectangular smoothing function and step function.

The signal in Figure 6 shows three distinct events. Taking the raw classifier output, it would be unclear if these were three portions of video where many animals are presented in quick succession or if there are truly three animals present and that the intermittent troughs are simply noise. By applying the smoothing function and rounding the smoothed values, a box is fitted around the three events confirming them as individual contigs, which matches the ground truth data. A rectangular smoothing function was chosen for its simplicity and low computational cost. The function was applied to the sequence twice to cheaply approximate triangular smoothing but was found to yield better results than a true triangular smoothing function because the different rectangular passes could be given varying parameters, allowing finer control.

Equation (1) calculates the mean of a single window and is repeated for each value in the signal, effectively sliding the window across the signal:

(1) $$f\left(n\right)=\frac{\sum_{i=n-\frac{1}{2}m}^{n+\frac{1}{2}m}S_{i}}{m}$$

where *m* is the window size, *n* is the output index and *S* is the array of input signal data. Indices that fall outside of *S* are taken as zero values. For a full pass over the signal, the function f(n) is repeated for all indices in *S*.

On some videos, the second smoothing and step function were found to be redundant, however, in cases where the positive samples lay particularly close to the start or end of the video, a second pass would often help to identify the contig boundaries more precisely. Choice of parameters for the smoothing function (window size) and step function (threshold) are highly dependent on the dataset and intended use-case. During development, factors such as video length, amplitude of classifier output, noisiness and distance between contigs were found to have an influence on the optimal parameters. The particular setup for our data was a smoothing pass with window size 20, followed by a threshold pass with threshold parameter 0.01, followed by a second smoothing pass with window size 10, followed by a final thresholding of 0.5. These values were chosen by trial and error whilst viewing visualisations of the signals and observing their effect. We do not claim that these values are optimal for the data but the results were found to be suitable for the use-case. In future, it may be more appropriate to have a subsystem learn the ideal values. However, given the low number of parameters in this case, manual experimentation was more time-efficient.

### 2.5. Frame Selection

In the previous section, ‘contig’ boundaries were derived where all of the frames in a single contig are associated with a particular animal. Here, the Frame Selector evaluates all frames that are within a contig, estimating the quality of the frame based on a number of factors (discussed later in this section). All frames that fall outside of the contig boundaries are discarded. After the Frame Selector model has made predictions for all of the relevant frames, the highest scoring frame for each contig (where frames are only compared against frames in their contig), is collected and sent to the next processing phase.

**Figure 7:**
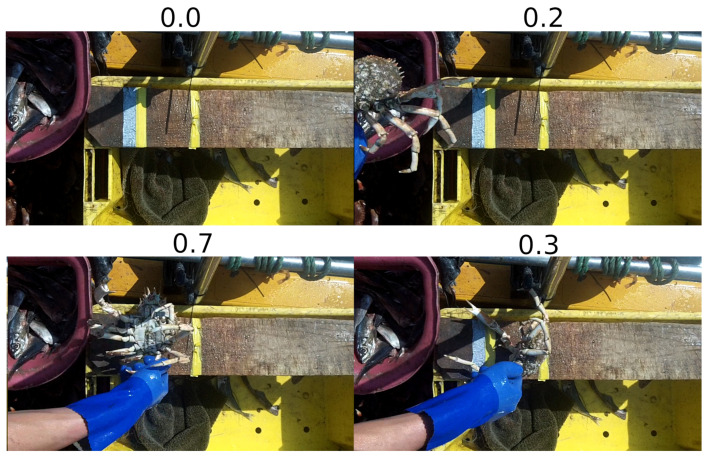
Example frames and their associated ‘representativeness’ scores.

Given that some of the animals must be viewed from multiple angles (e.g., lobsters), the Frame Selector system is comprised of multiple Convolutional Neural Networks (CNN) that each handle a different angle. The models take a single frame as input (rescaled to 320 × 180) and assign it a ‘representativeness’ score using a single output neuron. The architecture of each CNN is identical, however, they are trained on different datasets that label different views of the animals accordingly. Note that the images included in these various datasets are also identical and it is only the associated targets that vary. The target values ranged between 0 and 1, with a higher value corresponding to a more representative frame. The data was labelled by hand (by the researchers) with consideration made for the subject being relatively central in the frame, the subject’s distance from the camera, the orientation of the subject, the occlusion of the subject, motion blur, and overall image clarity. Cases where the subject is partially outside of the frame are labelled with an appropriately low score. Figure 7 shows a number of example frames and their corresponding scores given the previously mentioned criteria. Given the frames shown in Figure 7, the bottom-left frame would naturally be selected and used in the following stages. Whilst the bottom-right frame in Figure 7 is clear and the subject is central, the orientation (out-of-plane rotation) of the subject is not suitable for the following processing stages.

**Figure 8:**
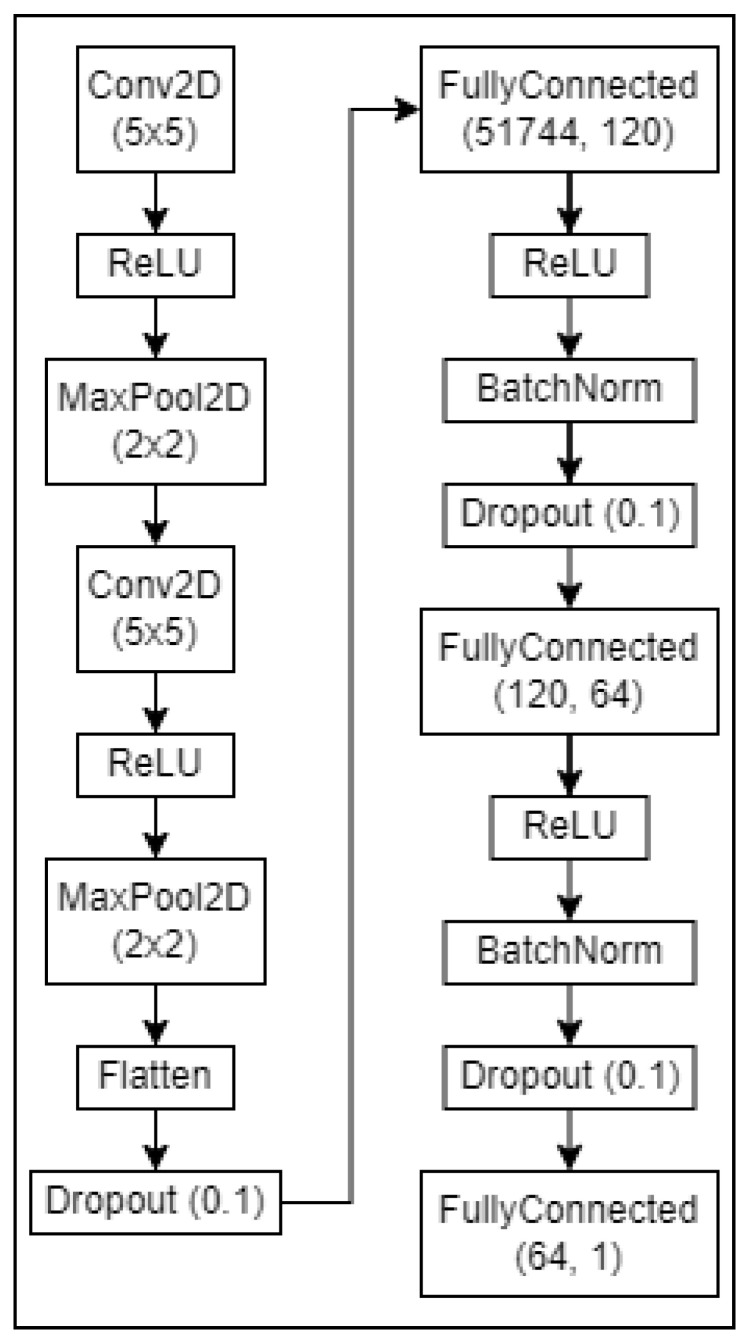
Architecture for a single CNN of the Frame Selector.

Much of the literature on Frame Selection recommends training a relative quality predictor [43,44,45] that compares two similar images and tasks the network with selecting the preferred image. This improves sample efficiency significantly because images can be used multiple times in different pairings to provide unique training inputs. However, this method of training only appears to perform well with sufficiently deep architectures and was found to be ineffective in an architecture appropriately lightweight for the low-power hardware. Instead, the much shallower architecture shown in Figure 8 is proposed to achieve reasonable inference time on the Raspberry Pi 3A+, trained with the traditional image X to target y mapping. Another factor that may be causing relative quality estimation to be ineffective is the low variance within a relatively small dataset, making differentiation between two samples a difficult task. The models (top and bottom) were each trained for 200 epochs using 950 images of animals presented at the corresponding angle. A learning rate of 1 × 10${}^{-5}$ was found to be optimal (for this volume of data).

For clarity, at inference time, the model will take each frame within a single contig (one at a time) and assign each one a score. The highest scoring frame in the contig will be taken as the ‘most representative’ frame of the animal (for that particular angle). This process is repeated for each contig where frames are only scored against frames in the same contig. At the end of this phase, one frame from each contig will have been collected for each angle (e.g., 5 contigs, 2 angles = 5 × 2 = 10 shortlisted frames). These frames will be used as input for the next phase.

### 2.6. Image Cropping and Keypoint Detection

As displayed in Figure 3, full-resolution versions (no downscaling) of each selected carapace frame (top view) are passed to an Object Detector. The role of this model is to fit a bounding box on the animal and crop the image to that size, removing excess background. As briefly discussed in Section 2.2, Keypoint Detection should be performed on true-scale images to maintain precision, because scaling or transforming the image in any way will introduce error into the inferred measurements. Whilst this means that downscaling is not possible, the image can still be cropped to greatly reduce the computational overhead without warping the image. YoloV5-small [46] was chosen as an implementation for Object Detection and has not been modified. This model achieves an inference speed of 0.85 fps on the Raspberry Pi 3A+, which is reasonable for this use-case considering that it will only need to process one image per animal. Note, also, that the images are cropped to a fixed size because the Keypoint Detector must have a consistent input shape. The crop size was determined by taking the average dimensions of the bounding boxes selected by the Object Detector when used on the dataset described in Section 2.4 without a fixed size. In the case of this work, the crop size was 560 × 540 but this depends entirely on the dataset used. The Object Detector also reports the class of object detected. Up to this stage in the pipeline, the models (Binary Classifier and Frame Selector) simply generalise across all animals as they are trained on mixed datasets. At this stage, however, it is important to know the class because different keypoints will be used to measure the different animals in the following phase.

**Figure 9:**
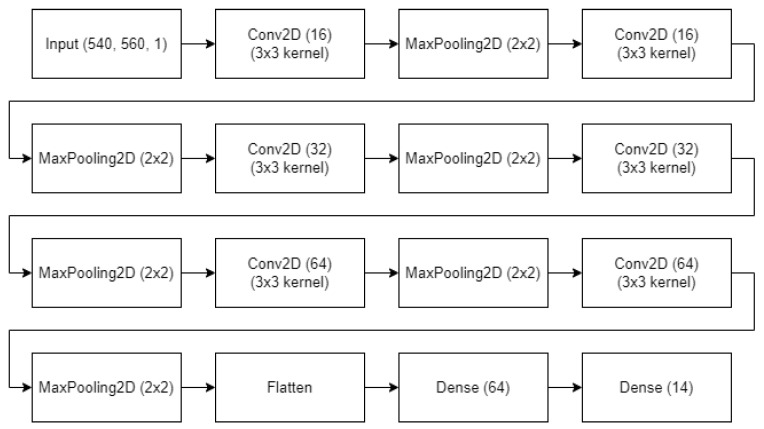
Architecture for the Keypoint Detector where the input size should correspond to the mean crop size found during the object detector stage.

The cropped frames can then be passed to the Keypoint Detector, for which the architecture is shown in Figure 9. The model has 6 alternating convolutional and max pooling layers before flattening to 1 dense layer and finally the output layer that contains 14 neurons, mapping to x and y values of the 7 named keypoints: ‘crab_left’, ‘crab_right’, ‘left_eye’, ‘right_eye’, ‘carapace_end’, ‘tail_end’ and ‘last_segment’. These keypoints were selected by experts in the field of marine science with the intention of estimating the age (correlated with the size) of each animal. The model used in this iteration of the pipeline was trained for 4k epochs using a learning rate of 1e-3, the Adam optimiser (keras) and Root Mean Squared Error (RMSE) as a loss function. The 1k samples were hand-labelled by the researchers using VIA [47].

## 3. Results

### 3.1. Model Performance

#### 3.1.1. Animal Segment Detection

**Figure 10:**
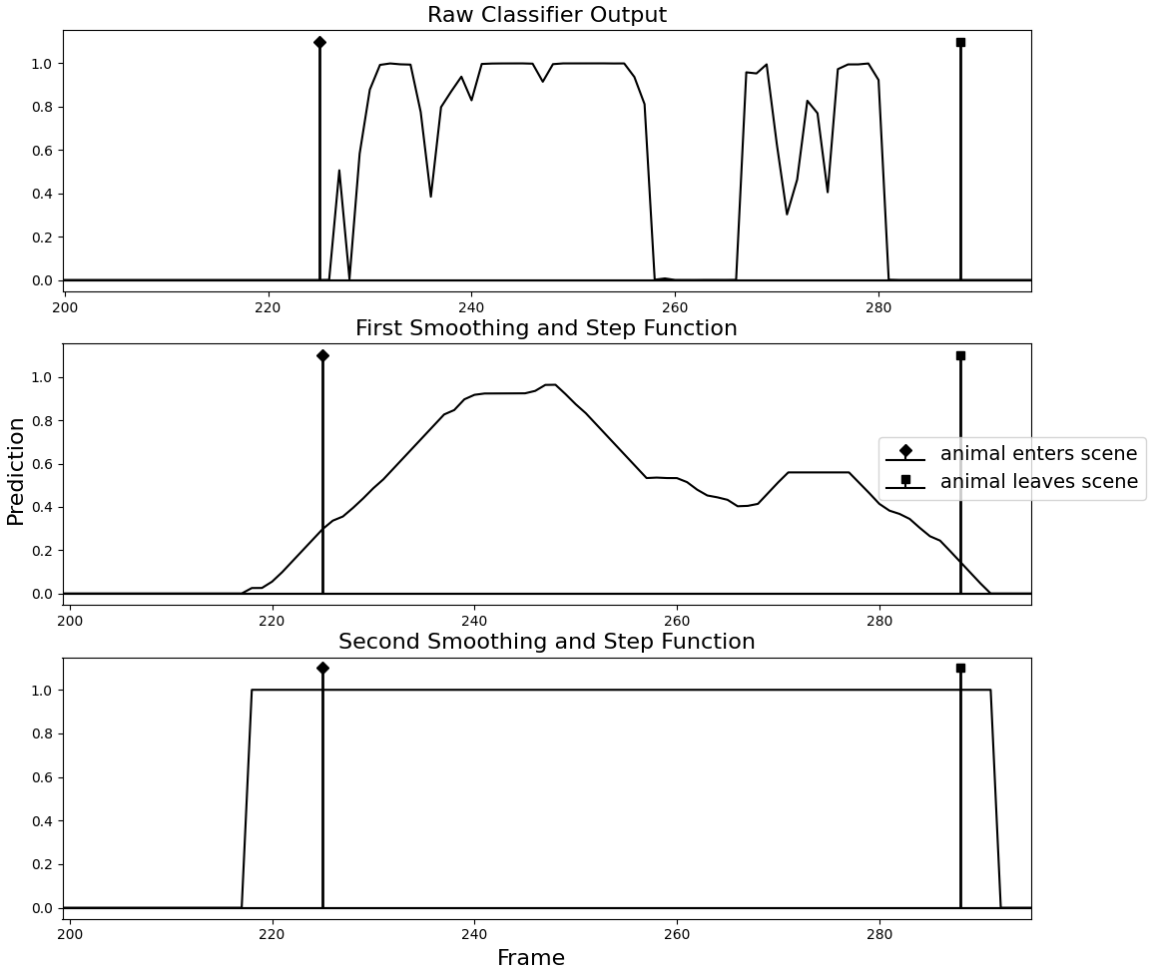
Smoothing function and step function applied to Binary Classifier predictions to approximate contigs. Diamond and square stem markers indicate the ground-truth timesteps at which subjects enter and leave the scene.

The goal of this stage is to identify the start and end of a region that features a particular animal based on the noisy and volatile prediction signal of the lightweight classification model defined in Section 2.4. Figure 10 shows the smoothing technique applied to a test video, previously unseen by the classifier. Note that the approximated contig is typically larger than the ground truth region. This behaviour is caused by the choice of window size, a parameter of the smoothing function. Setting a smaller window size would cause the smoothed curve to react to sharp changes more quickly. However, it was found that oversized contig boundaries were beneficial as it ensured that all animal frames would be collected.

Whilst it is difficult to evaluate this processing phase quantitatively, the goal of approximating contig boundaries on test data was achieved with a 90% success rate on average, where ‘success’ is when a contig encompasses exactly one animal. On the test videos used, 1/10 animals were not detected at all, though this may be due to them appearing in the scene for a shorter period of time than average, causing the signal to be rounded down. Whilst moving the subject too quickly or presenting the subject for too short a time can result in animals being missed, the tests performed suggested that the method is robust against false positives and very rarely over-counted. This implies that there is an upper bound to the sorting speed (of the fishers) that the system can handle, but there is no lower bound. One other failure case observed during testing was when two animals were presented in quick succession, without a sufficient gap in between, causing a single contig to span over both. This meant that only one of them would be counted and there is no guarantee that the selected frames of different angles will correspond to the same animal.

#### 3.1.2. Frame Selection

As discussed in Section 2.5, relative quality estimation was not found to be effective for training the Frame Selectors in this scenario. For testing, however, presenting two frames to the model and recording whether it predicted a higher score on the better frame was found to be a good analogue for the use-case. This is because, at runtime, predicting the precise quality of a frame is not important, so long as it awards a higher score to the better frame. Evaluating the models in this way does not penalise them for distance from the ground truth quality as many metrics would. Using this method, the ‘top’ and ‘bottom’ Frame Selectors achieved 76% and 78% accuracy (selected the better image), respectively, on the test data.

#### 3.1.3. Object Detection

**Figure 11:**
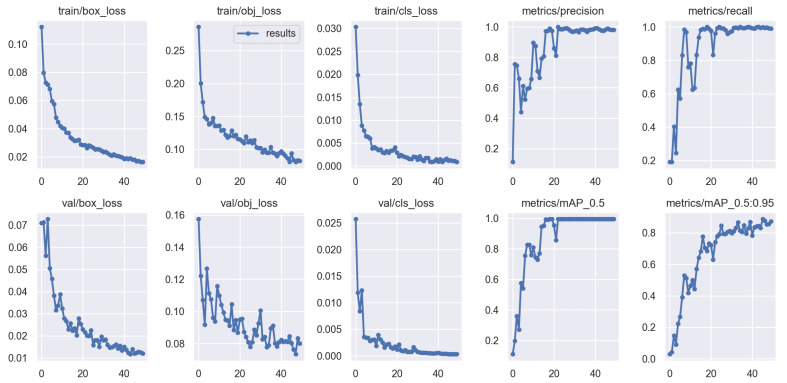
Object Detector training metrics over 50 epochs.

**Figure 12:**
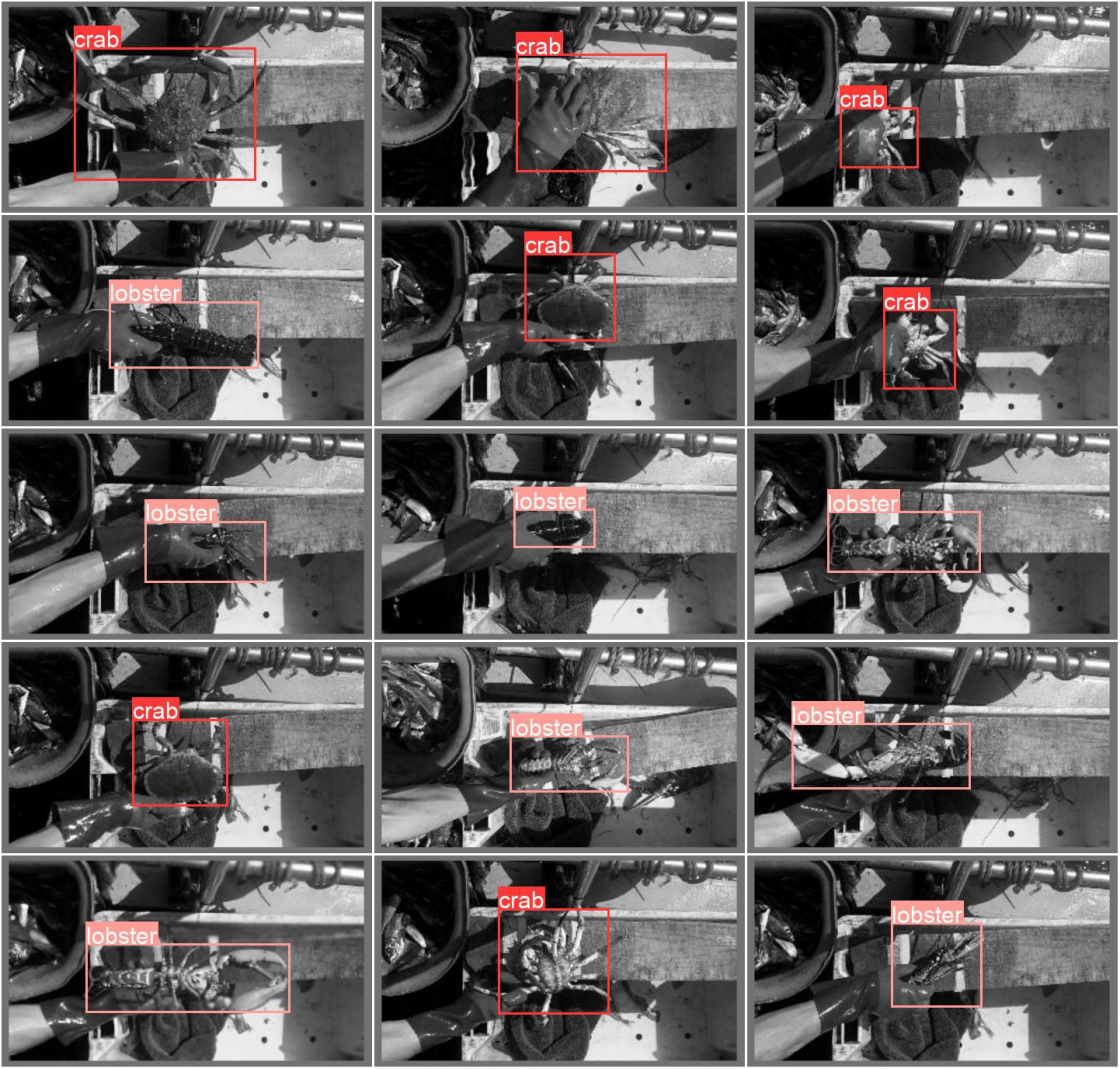
Object Detector inference on a batch of test samples.

The training metrics of the Object Detector are shown in Figure 11. After training was complete (50 epochs), the mAP@[0.5] and mAP@[0.5:0.95] (step size 0.01) [46,48] were 0.995 and 0.873, respectively. Despite the data having a severe animal–class imbalance, class overfitting was not observed. The model was then tested on 50 unseen samples (Figure 12 shows an example batch) and achieved a mAP@[0.5:0.95] of 0.859. The very minor shortfall between training and testing mAP confirms that the model is not overfitting the training data. Note that the training and test datasets were comprised solely of frames that contain animals, however, the datasets were a mixture of high and low quality frames. The results shown here demonstrate the performance in the worst case (using low quality frames) when, in practice, the pipeline will pass only the highest quality frames available. This implies that the model may perform better than these results on average when used as part of the pipeline.

#### 3.1.4. Keypoint Detection

**Figure 13:**
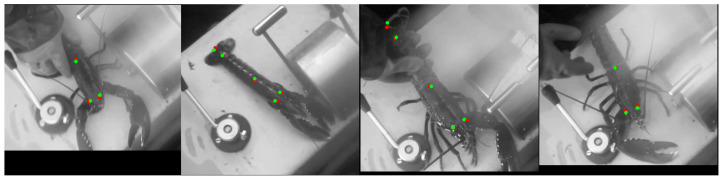
Keypoint Detector inference on test data. Red markers show keypoints predicted by the model and Green markers show ground-truth keypoints.

Figure 13 shows that the keypoint detector is correctly approximating the locations of the keypoints. The model achieved a Mean Euclidean Error of 28.09 pixels, corresponding to a 5.76% error given the size of the animals within the image on average. These results are taken from 32 test samples. Considering that only a small portion of data has been labelled for keypoints at this stage, and that the annotated data are heavily imbalanced in terms of animal class and are primarily from a single location, the results are promising. The low variance in this data has been somewhat mitigated by applying the augmentation techniques described in Section 2.3, although the generalisation ability of the model was still found to be lacking for certain test samples. It is believed that additional data being collected in future will improve this model significantly.

### 3.2. Overall Pipeline

**Figure 14:**
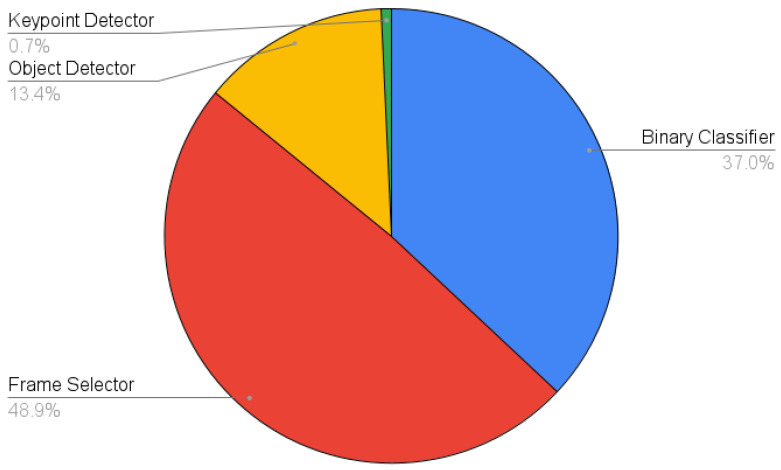
Computation time breakdown for each model in the pipeline.

Considering that computational efficiency was paramount in this work given the hardware limitations, a runtime analysis of the system was conducted. Figure 14 shows the portion of runtime each model used when processing a single video from start to finish. Note that the Binary Classifier and Frame Selector are not slower than the Object Detector and Keypoint Detector because the latter two are only invoked for one frame per animal, whereas the Binary Classifier and Frame Selector are invoked on all frames or all in-contig frames, respectively. Whilst the Frame Selector is not processing every single frame, as with the Binary Classifier, quality estimation is a more nuanced and complex task than classification. Given that the Frame Selector still processes a significant number of frames (∼40% in our experiments), it is natural that it requires the most processing time. Table 2 shows a more detailed breakdown of the processing time for each stage and the number of frames being processed in that time. These results further support the argument for combining a weak yet lightweight classification model and a more complex object detection model that is only invoked when necessary, as using the Object Detector alone would be unfeasibly slow in the target hardware.

## 4. Discussion

Section 3 has presented an evaluation of each component in the proposed pipeline and demonstrates the performance of the system as a whole. These findings show that the system is already performing well and highlights that the most important aspect moving forward is data acquisition. Additional data are likely to improve the accuracy and, more importantly, the generalisation (different lighting conditions, backgrounds, camera setups, etc.) ability of each model. The Keypoint Detector has been the most difficult to train and the dataset used for this model exhibited severe class (animal) imbalance that was unavoidable at this stage. This will be addressed in future iterations of the work and is expected to have a positive impact on performance. With regards to efficiency, the pipeline is still being developed and it is likely that further optimisations can be made to improve the overall runtime. The Frame Selector will be the most heavily scrutinised as ∼50% of the pipeline runtime is spent on this phase (see Figure 14). Model distillation and quantization [49,50] will also be investigated as methods of improving inference time.

Taking a broad view of the pipeline as a whole, it may appear that the Binary Classifier and Frame Selector at the beginning of the pipeline are functionally redundant and can be subsumed by the Object Detector. This is theoretically true, however, using the Object Detector on every single frame would be extremely inefficient as it is a much more complex and computationally expensive model. On the target hardware, MobileNetV3-small runs between 9.43 fps and 11.99 fps, whereas YoloV5 was found to run at ∼0.85 fps. From this, it becomes obvious that running every frame through the Object Detector would take significantly longer. The Binary Classifier, smoothing techniques and Frame Selector fulfill the task well for a fraction of the cost and, in this proposed system, the Object Detector only needs to infer one frame per animal.

The application of this automated data collection could revolutionise the ability to gather catch composition data that have sufficient spatial and temporal coverage to inform stock assessment in these important fisheries. With a sentinel fleet, there is the opportunity for monthly or more frequent data that represent the full spatial extent of the fishery and the full diversity of the fleet, providing high-quality, rich data to inform a range of potential stock assessment methodologies. Whilst this piece of work has focused on two species of commercial importance in European waters, the concept and pipeline could be applied to other fisheries globally where catch is handled in such a way that the animals can be captured on the video.

## 5. Conclusions

This work has investigated the feasibility of a Computer-Vision-based animal counting and measurement system on low-powered hardware such as a Raspberry Pi 3A+. The prototype pipeline described in this work acts as a proof of concept and is a promising foundation that will be built upon, as described in Section 4. The work will also be extended to address the photogrammetry problem by inferring measurements based on the predicted keypoints. Another aspect left to future work is the problem of automatically sexing the animals. This will use the underside frames gathered at the Frame Selector phase of the pipeline and will be fed into a simple binary classifier trained on images labelled by sex.

## Figures and Tables

**Table 1:** Candidate Binary Classification models compared by memory usage and speed. OOM denotes that the model crashed during inference due to lack of memory in the target hardware.

Model	Memory Usage (GB)	Inference Time (s)	FPS	OOM
MobileNet-V1	5.5	n/a	n/a	Yes
TripleNet-S	1.5	20.132	0.050	Yes
EfficientNetB0	0.24	2.182	0.458	No
MobileNetV3-small	0.43	0.102	9.804	No

**Table 2:** Each component of the pipeline compared by processing time used and the number of frames processed in that time.

Component	Seconds of Processing	Frames Processed	FPS
Binary Classifier	300.02	3600	11.998
Frame Selector	410.54	1486	3.619
Object Detector	109.48	15	0.137
Keypoint Detector	5.71	15	2.626

## Data Availability

Sample data and software source code will be made available on acceptance.

## Abbreviations

The following abbreviations are used in this manuscript:

MSY	Maximum Sustainable Yield
FMP	Fisheries Management Plans
SOTA	State of the Art
NAS	Neural Architecture Search
SBC	Single Board Computer
GSM	Global System for Mobile Communication
GPS	Global Positioning System
GNSS	Global Navigation Satellite System
PCB	Printed Circuit Board
UPS	Uninterruptible Power Supply
FPS	Frames Per Second
CNN	Convolutional Neural Network
RMSE	Root Mean Squared Error
ROI	Region of Interest
